# Association between the ascending aortic length and sporadic type A aortic dissection

**DOI:** 10.3389/fcvm.2025.1684930

**Published:** 2026-02-04

**Authors:** Dan Rong, Shaofan Wang, Feng Liu, Jianhan Yin, Wei Guo

**Affiliations:** 1 Department of Vascular and Endovascular Surgery, Chinese PLA General Hospital, Beijing, China; 2 School of Medicine, Nankai University, Tianjin, China; 3 Chinese PLA Medical School, Beijing, China

**Keywords:** ascending aortic length, Chinese population, predictive factor, ROC (receiver operating characteristic curve), type A aortic dissection

## Abstract

**Objective:**

This study was performed to outline the aortic dimensions of type A aortic dissection and to investigate the association between the ascending aortic length and sporadic, non-syndromic type A aortic dissection in a Chinese population.

**Methods:**

In this cross-sectional study, all consecutive patients diagnosed with type A aortic dissection between January 2016 and December 2020 were identified. Parameters were measured based on the centerline method. The predissection aortic diameter and length were calculated. Multivariate logistic regression models were used to assess the association between the ascending aortic length and type A aortic dissection. A receiver operating characteristic analysis was performed to calculate the area under the curve and optimal cutoff value.

**Results:**

A total of 146 patients and 146 propensity score-matched controls were included in the study. The median length of the ascending aorta was 21 mm longer in the patient group than in the control group (median, 110.0 mm vs. 89.0 mm), and was still 15.4 mm longer after adjusting for the predissection length (median, 104.4 mm). The ascending aortic length was independently associated with type A aortic dissection (odds ratio, 5.34; 95% confidence interval, 3.59–7.92). The ascending aortic length revealed a larger area under the curve, and an optimal cutoff value of 9.8 cm was revealed.

**Conclusion:**

The ascending aortic length (AAL) appears to be associated with type A aortic dissection (TAAD) and may complement the aortic diameter in morphologic risk assessment, but additional prospective studies with standardized measurements and body-size adjustment are required before clinical thresholds can be established. Observed AAL values associated with TAAD were lower in this Chinese cohort than in the Western population.

## Highlights

- The ascending aortic length is more promising as a predictive factor of type A aortic dissection than diameter, with the intervention threshold in the Chinese population different from that in the Western population.
- The aortic diameter is insufficient to identify candidate patients at high risk of type A aortic dissection (TAAD) for prophylactic surgery. Recently, the ascending aortic length (AAL) has emerged to demonstrate the potential relationship with TAAD. Further evaluation of AAL as a predictive factor for TAAD is required, especially in different races of the population.

## Introduction

Acute type A aortic dissection (TAAD) is a catastrophic disease with a mortality rate of 50% within the first 48 h and 90% within the first month if not treated (1). Prophylactic surgery is a recommended therapy for patients at high risk of TAAD to prevent sudden death from dissection onset (1). The best-established morphologic predictors of TAAD are the maximal diameter and dilation rate of the ascending aorta, considered alongside clinical risk factors such as family history and heritable aortopathies. Patients with an ascending aortic diameter of >60 mm have a markedly higher incidence of dissection and rupture (2). Therefore, current guidelines recommended a prophylactic operation for patients with an ascending aortic diameter of ≥55 mm. However, diameter alone is insufficient to identify candidate patients for prophylactic ascending aortic replacement. The International Registry of Acute Aortic Dissection (IRAD) study showed that approximately 60% of patients with TAAD have an ascending aortic diameter of <55 mm (3). When modeling the dissected ascending aorta to its predissection dimension, this threshold fails to be met in 97% of patients (4). Thus, an investigation of additional risk factors for TAAD is warranted to better identify candidate patients for prophylactic surgery.

Rylski et al. (5) demonstrated an average increase of 31.9% in diameter and 5.4% in length in the ascending aorta after the occurrence of dissection. The ascending aortic length (AAL) has slighter variation than the aortic diameter when the aorta is dissected and is another potential risk factor for TAAD. A few studies have illustrated the potential association between an increased AAL and a higher incidence of TAAD in the Western population (6–9). The aortic dimensions and demographic characteristics of aortic dissection are different between Western and Chinese populations. The present study was performed to outline the aortic dimensions of TAAD and investigate the potential association between the AAL and TAAD in a Chinese population.

## Methods

### Study design and population

This cross-sectional study was designed to evaluate the potential relationship between the AAL and TAAD. The protocol of this study was registered with ClinicalTrials.gov (ChiCTR-POC-17011726) and approved by the Institutional Review Board of Chinese PLA General Hospital. The requirement for informed consent was waived because of the retrospective observational nature of the study. This study was reported in accordance with the STROBE statement.

All consecutive patients admitted to our institution between January 2016 and December 2020 with TAAD, diagnosed by computed tomography angiography (CTA), were eligible for the present study. Patients with a history of diagnosed connective tissue disease (Marfan syndrome, Loeys–Dietz syndrome, Ehlers–Danlos syndrome, or Turner syndrome), a family history of aortic disease (aneurysm or dissection), a bicuspid aortic valve, and previous aortic surgery were excluded. Patients were diagnosed with TAAD if the dissection involved the ascending aorta.

During the study period, 190 patients were diagnosed with TAAD in our hospital. Of these 190 patients, 178 had qualified CTA images. Of these 178, 23 were diagnosed with connective tissue disease, four had a bicuspid aortic valve, and five had previous aortic surgeries (three aortic valve replacements and two thoracic endovascular aortic repairs). After exclusion of these patients, a total of 146 patients were included in the study as the patient group for image measurement and data analysis.

Propensity score matching was applied to acquire a balanced control group from all patients (*n* = 1,193) who underwent a CTA examination and in whom aortic disease was ruled out during the same period. The propensity score was calculated by a logistic regression model. Age, sex, height, weight, and date of examination were included as covariates in the model to further reduce potential confounding. Patients with TAAD were matched in a 1:1 ratio to control participants based on the propensity score using a caliper width of 0.2.

### Image measurement

The CTA images were anonymized before measurement to reduce observation bias. The 3mensio Workstation version 10.2 (3 mensio Medical Imaging B.V., Maastricht, Netherlands) was used to measure the CTA images. These images were assigned to two independent specialists (DR and LF) for measurement. The interobserver and intraobserver measurement agreements were assessed by performing a Bland–Altman analysis.

The aortic annulus plane was defined by three markers at the cusp nadirs of the sinus of Valsalva. Diameters were measured at planes perpendicular to the centerline. Lengths were measured at the centerline-based stretched plane. Multicollinearity among the continuous predictor variables was assessed using variance inflation factors (VIFs). A VIF value of less than 5 was considered to indicate the absence of severe multicollinearity.

### Calculated predissection diameters

Recent studies have proven that ascending aortic dimensions significantly change immediately after dissection (4, 10). Rylski et al. *(*4) analyzed the largest cohort of patients (*n* = 63) with TAAD who also had CTA images before aortic dissection, demonstrating a 31.9% increase in the diameter of the middle ascending aorta and a 5.4% increase in the AAL after dissection. To determine whether the AAL and diameter are risk factors for TAAD, we used the equation D1 = (1 + 31.9%) × D2 to calculate the predissection diameter, where D1 is the postdissection diameter and D2 is the predissection diameter. Similarly, we used the equation L1 = (1 + 5.4%) × L2 to calculate the predissection length, where L1 is the postdissection length and L2 is the predissection length.

### Statistical analysis

Multiple imputation was conducted for missing data on height and weight (five patients with TAAD and three controls). Five datasets were imputed and the results pooled according to Rubin's rules (11). Categorical variables were presented as frequency and percentage. Continuous variables were presented as median with first and third quartiles. Participant demographics and the aortic dimensions of the patients with TAAD and control participants were compared using the Mann–Whitney test for continuous variables and the chi-square test for categorical variables.

Initially, the association between the AAL and TAAD was assessed using a univariate logistic regression model. We then used change-in-estimate criteria to select covariates. Variables deemed as clinically related to the AAL were also included as covariates. Selected covariates were introduced in a multivariate logistic regression model to further assess the association between the AAL and TAAD. The unit of the AAL was transformed from millimeter to centimeter in the logistic regression analysis for better applicability when interpreting the analysis results. Unadjusted and adjusted odds ratios (ORs) with 95% confidence intervals (CIs) were calculated. Interaction and stratified analyses were then performed according to the variables used in the propensity score matching [age grouping, sex, height, weight, and body surface area (BSA)] to validate the robustness of the results. Finally, a receiver operating characteristic (ROC) analysis was performed to calculate the area under the curve (AUC) and optimal cutoff value of the AAL. The cutoff point on the ROC curve closest to the top left part was used.

All *P*-values were two-tailed, and a *P*-value of <0.05 was considered statistically significant. All statistical analyses were conducted using the statistical software package R (http://www.R-project.org, The R Foundation) and Free Statistics software version 1.3 (http://www.clinicalscientists.cn/freestatistics/).

## Results

### Participant demographics and aortic dimensions

The data analysis included 146 patients with TAAD and 146 propensity score-matched control participants. Demographic data and aortic dimension data are presented in Table 1. There was no difference in age between the patient group [median age, 56.5 years; interquartile range (IQR), 50–65 years] and the control group (median age, 57 years; IQR, 50–64 years). In both groups, male sex was predominant (67.8%). Weight (median, 73 kg; IQR, 65–82 kg vs. median, 73 kg; 64.2–78 kg) and height (median, 170 cm; IQR, 163.1–175 cm vs. median, 169 cm; IQR, 164–173 cm) were also well matched. The arch type was also not significantly different between the two groups.

**Table 1:** Patient demographics and aortic dimensions.

Characteristics	Total	Patients with TAAD (*n* = 146)	Control patients (*n* = 146)	*P*-value
Age (years)	57.0 (50.0, 65.0)	56.5 (50.0, 65.0)	57.0 (50.0, 64.0)	0.942
Male (%)	198 (67.8)	99 (67.8)	99 (67.8)	1
Weight (kg)	73.0 (65.0, 80.0)	73.0 (65.0, 82.0)	73.0 (64.2, 78.0)	0.374
Height (cm)	170.0 (163.4, 174.0)	170.0 (163.1, 175.0)	169.0 (164.0, 173.0)	0.477
BSA	1.8 (1.7, 1.9)	1.8 (1.7, 2.0)	1.8 (1.7, 1.9)	0.361
Hypertension (%)	206 (70.5)	107 (73.3)	99 (67.8)	
Smoking history (%)	128 (43.8)	61 (41.8)	67 (45.9)	
Dyslipidemia (%)	144 (49.3)	41 (28.1)	103 (70.5)	
Diabetes mellitus (%)	48 (16.4)	9 (6.1)	39 (26.7)	
Arch type				0.202
Type I	157 (53.8)	74 (50.7)	83 (56.8)	
Type II	54 (18.5)	28 (19.2)	26 (17.8)	
Type III	81 (27.7)	44 (30.1)	37 (25.3)	
Bovine arch	35 (12.0)	20 (13.7)	15 (10.3)	0.165
Diameter of aortic annulus	26.6 (25.0, 28.5)	26.9 (25.0, 29.2)	26.4 (25.0, 28.1)	0.041
Diameter at STJ	34.4 (30.0, 44.6)	44.6 (40.1, 48.0)	29.9 (28.2, 31.9)	<0.001
Diameter at BCT	37.5 (33.1, 43.4)	43.4 (40.1, 47.1)	33.2 (30.8, 35.1)	<0.001
Diameter at middle ascending aorta	40.3 (34.7, 48.9)	48.9 (45.7, 53.4)	34.7 (32.8, 36.8)	<0.001
Maximum diameter >55 mm	33 (11.3)	33 (22.6)	0 (0.0)	<0.001
Predissection diameter at middle ascending aorta	NA	37.0 (34.6, 40.5)	NA	<0.001
Maximum predissection diameter >55 mm	1 (0.3)	1 (0.7)	0 (0.0)	1
AAL	98.0 (89.0, 110.0)	110.0 (102.0, 120.2)	89.0 (84.0, 95.0)	<0.001
Predissection AAL	NA	104.4 (96.8, 114.1)	NA	<0.001
Tubular ascending aortic length	70.0 (62.0, 80.0)	80.0 (72.5, 90.0)	63.0 (58.5, 70.0)	<0.001
Height of sinus of valsalva	27.0 (24.6, 29.4)	28.4 (26.0, 33.9)	25.0 (24.0, 27.8)	<0.001

BSA, body surface area; STJ, sinotubular junction; BCT, brachiocephalic trunk; AAL, ascending aortic length.

**Figure 1:**
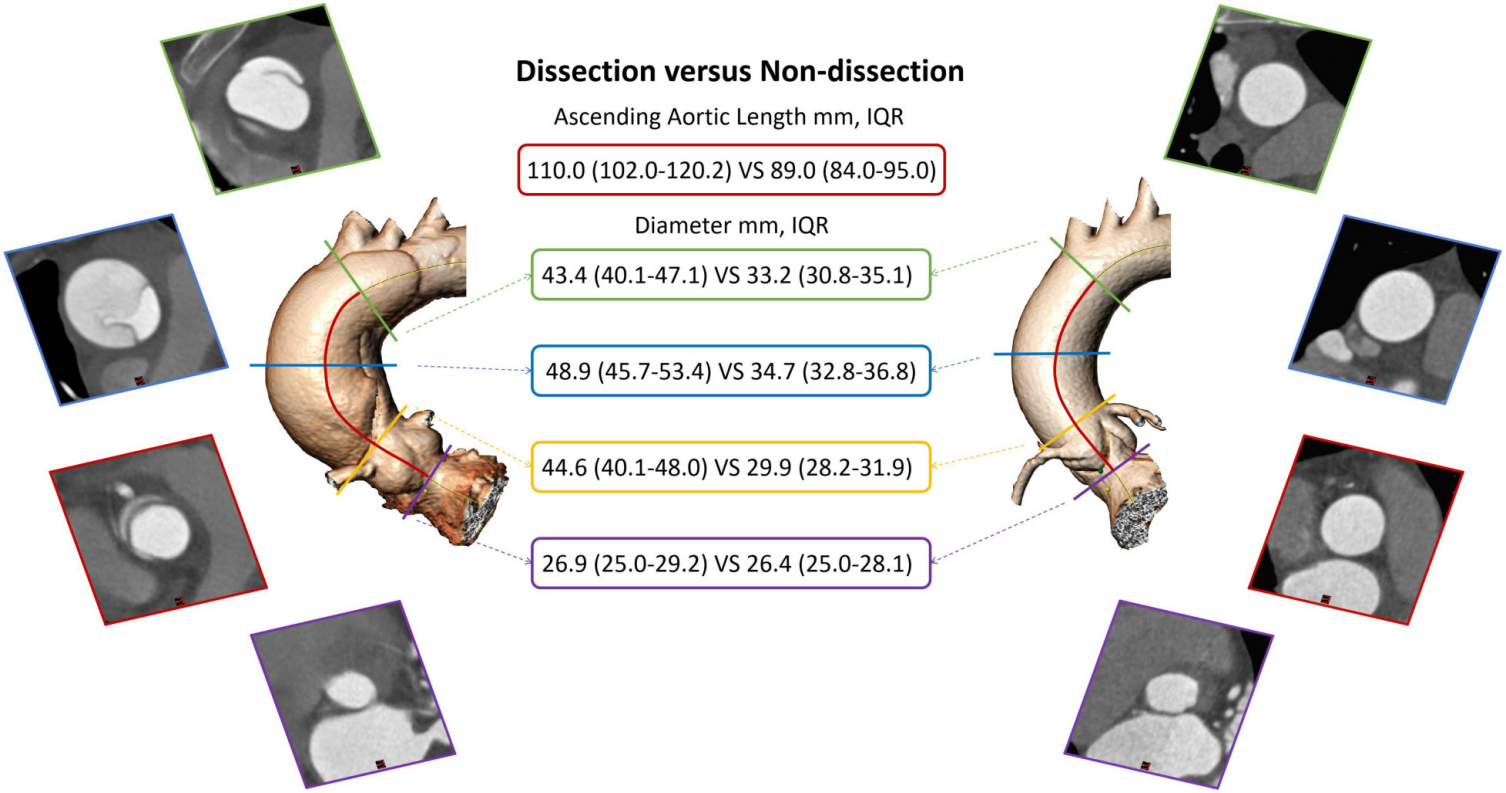
Ascending aortic dimension comparison between dissected and non-dissected aorta. IQR, interquartile range.

The diameter of the aortic annulus was almost the same in the two groups (median, 26.9 mm; IQR, 25–29.2 mm vs. median, 26.4 mm; IQR, 25–28.1 mm), but the difference was still statistically significant. The diameters of the STJ, BCT, and middle ascending aorta were significantly larger in the patient group (Figure 1), even when transformed to the predissection diameter at the middle ascending aorta. Only 22.6% patients with TAAD presented with an aortic diameter >55 mm (Table 1).

The median AAL was 21 mm longer in the patient group than in the control group (Figure 1), and it was still 15.4 mm longer after making adjustments to the predissection AAL. Other variables of length, including the tubular AAL and height of the sinus of Valsalva, were also significantly longer in the patient group (Table 1).

**Figure 2:**
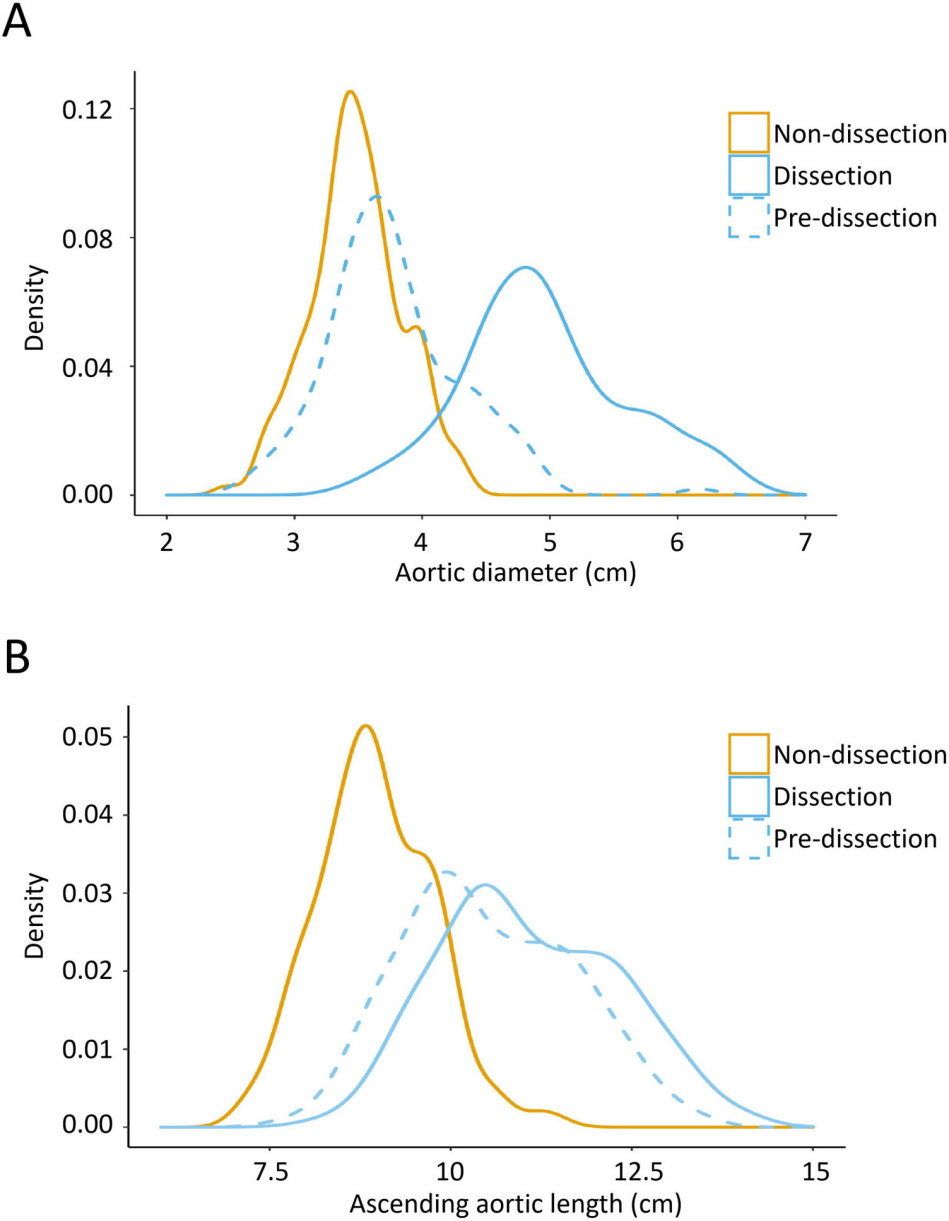
Kernel density plots demonstrating the distribution of the aortic diameter **(A)** and AAL **(B)** in the TAAD group and control group. The peaks in this figure display the concentration situation of the values in the aortic diameter and AAL. TAAD, type A aortic dissection; AAL, ascending aortic length.

The distribution of the aortic diameter and the AAL is illustrated in Figure 2. A significant “leftward shift” of both the aortic diameter and the AAL distribution was observed in the patient group when modeling for predissection dimensions (*P* < 0.001). A larger “leftward shift” of the aortic diameter was observed compared with the AAL, indicating that the aortic diameter had a more significant variation after dissection onset.

**Figure 3:**
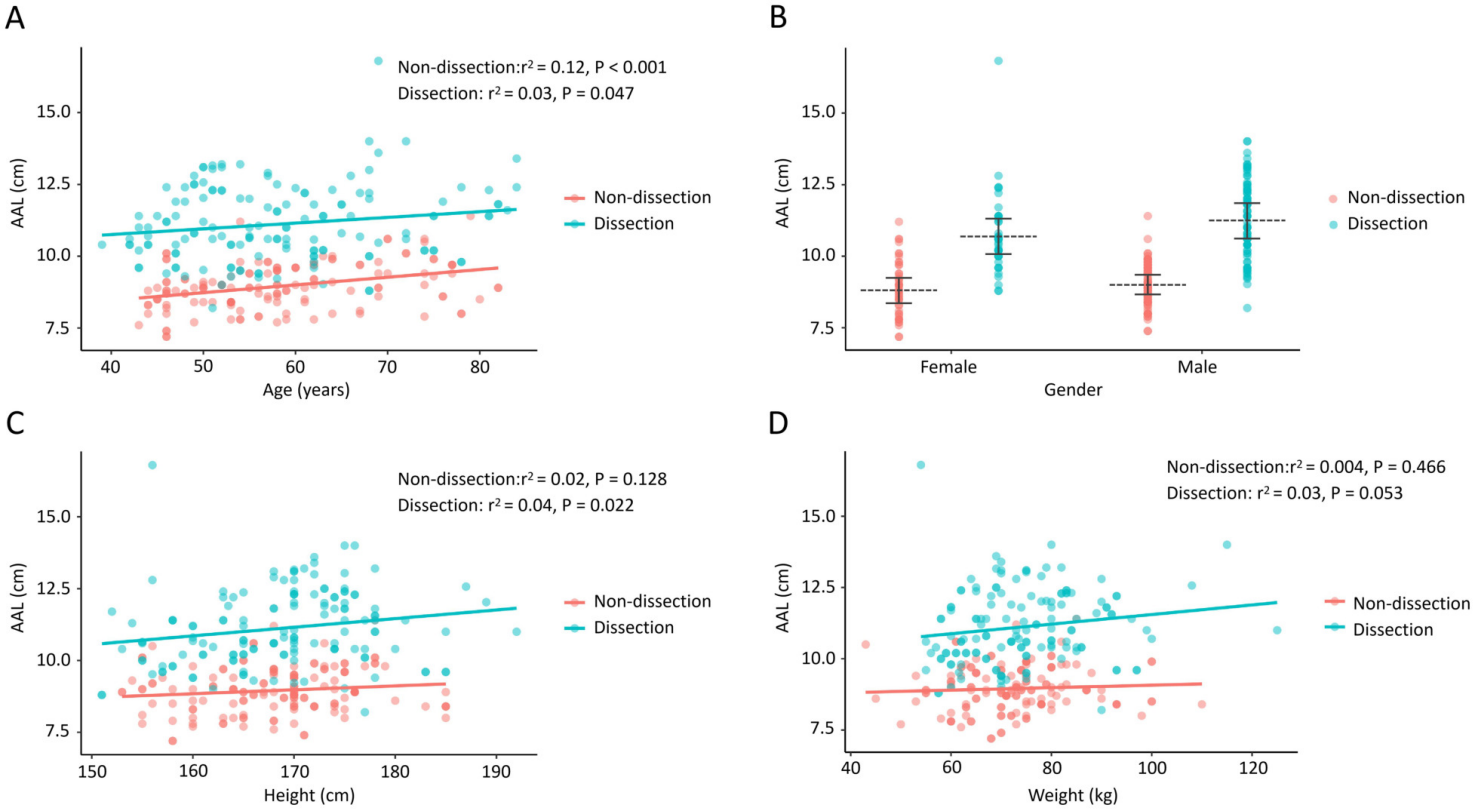
The association of the AAL with age, gender, height, and weight in TAAD patients and control participants. **(A)** The AAL positively correlates with increasing age in both the TAAD group and the control group. **(B)** Patients with TAAD presented with a longer AAL in both males and females. **(C)** The AAL correlates with height in the TAAD group. **(D)** The AAL does not correlate with body weight in both groups. AAL, ascending aortic length; TAAD, type A aortic dissection.

The distribution of the AAL in age intervals, different sexes, and height and weight intervals from the two groups is presented in Figure 3. Increased age was associated with a longer AAL in both patient (*r*^2^ = 0.03, *P* = 0.047) and control (*r*^2^ = 0.12, *P* < 0.001) groups (Figure 3A). Patients with TAAD had a significantly longer ascending aorta than controls, and male patients had a longer ascending aorta than their female counterparts (Figure 3B). Increasing height had minimal correlation with the AAL in only patients with TAAD (*r*^2^ = 0.04, *P* = 0.022) (Figure 3C). Weight had no correlation with the AAL in both groups (Figure 3D).

### Association between the AAL and TAAD

The univariate logistic regression analysis demonstrated a significant association between the AAL and TAAD (OR, 5.34; 95% CI, 3.59–7.92) (Table 2). We included the parameters of age, sex, height, weight, BSA, and predissection aortic diameter in the selection of covariates. Based on the change-in-estimate criteria, none of the abovementioned variables was selected as a covariate. Considering that the AAL was associated with age, sex, height, arch type and aortic diameter, we still included these variables as covariates in the multivariate logistic regression analysis. After adjusting for age, sex, height, and arch type in model 1, we found that the association between the AAL and TAAD was still statistically significant (OR, 6.98; 95% CI, 4.4–11.09) (Table 2). Further adjustment for the aortic diameter in model 2, which was closely related to the AAL, did not attenuate the association of the AAL and TAAD (OR, 7.93; 95% CI, 4.75–13.26) (Table 2). In addition, in model 3, further adjustments for hypertension, smoking history, dyslipidemia, and diabetes mellitus also yielded a significant association (OR, 7.34; 95% CI, 4.19–12.88).

**Table 2:** Multivariate logistic regression models evaluating the association between the AAL and TAAD.

Variable	Study participants			OR (95%CI)		
	Cases	Controls	Crude	Model 1^a^	Model 2^b^	Model 3^c^
AAL (cm)	146	146	5.34 (3.59, 7.92)	6.98 (4.40, 11.09)	7.93 (4.7, 13.26)	7.35 (4.19, 12.88)

a Adjusted for age, sex, height, and arch type.

b Adjusted covariates in model 1 plus predissection diameter at the middle ascending aorta.

c Adjusted covariates in model 2 plus hypertension, smoking history, dyslipidemia, and diabetes mellitus.

AAL, ascending aortic length; TAAD, type A aortic dissection; OR, odds ratio; CI, confidence interval.

The results of the sensitivity analysis (i.e., interaction and stratified analyses) are presented in Table 3. The association of the AAL and TAAD was statistically significant in both the younger group (adjusted OR, 12.85; 95% CI, 5.4–30.58) and the older group (adjusted OR, 4.4; 95% CI, 2.42–8) and in both male participants (adjusted OR, 12.64; 95% CI, 6.11–26.17) and female participants (adjusted OR, 2.92; 95% CI, 1.44–5.92). The association of the AAL and TAAD was statistically significant in each height, weight, and BSA grouping after adjustments were made for the arch type, the aortic diameter, and other stratification factors. The interaction analysis revealed no interactive role in the association between the AAL and TAAD.

**Table 3:** Multiple logistic regression models evaluating the association between the AAL and TAAD according to baseline characteristics.

Subgroup	Study participants		OR (95% CI)		*P*-value for interaction
	Cases (*N* = 146)	Controls (*N* = 146)	Crude	Mutually adjusted^a^	
Age					0.347
<57	73 (51.0)	70 (49.0)	7.57 (3.84, 14.92)	12.85 (5.4, 30.58)	
≥57	73 (49.0)	76 (51.0)	5.20 (2.99, 9.05)	4.40 (2.42, 8.00)	
Sex					0.345
Male	99 (50.0)	99 (50.0)	6.46 (3.83, 10.91)	12.64 (6.11, 26.17)	
Female	47 (50.0)	47 (50.0)	4.00 (2.17, 7.37)	2.92 (1.44, 5.92)	
Height					0.830
<170	70 (48.6)	74 (51.4)	4.96 (2.84, 8.68)	4.67 (2.43, 8.97)	
≥170	76 (51.4)	72 (48.6)	6.22 (3.47, 11.16)	17.81 (6.7, 47.37)	
Weight					0.573
<73	71 (49.7)	72 (50.3)	4.81 (2.77, 8.36)	4.41 (2.28, 8.53)	
≥73	75 (50.3)	74 (49.7)	6.70 (3.65, 12.3)	13.21 (5.7, 30.65)	
BSA					0.946
<1.8	67 (47.9)	73 (52.1)	5.39 (2.98, 9.74)	4.74 (2.33, 9.64)	
≥1.8	79 (52.0)	73 (48.0)	5.71 (3.28, 9.97)	10.28 (4.88, 21.67)	

OR, odds ratio; CI, confidence interval; BSA, body surface area; AAL, ascending aortic length; TAAD, type A aortic dissection.

a Each stratification adjusted for arch type, predissection diameter at the middle ascending aorta, and all stratification factors (age, sex, height, weight, and BSA) except itself.

**Figure 4:**
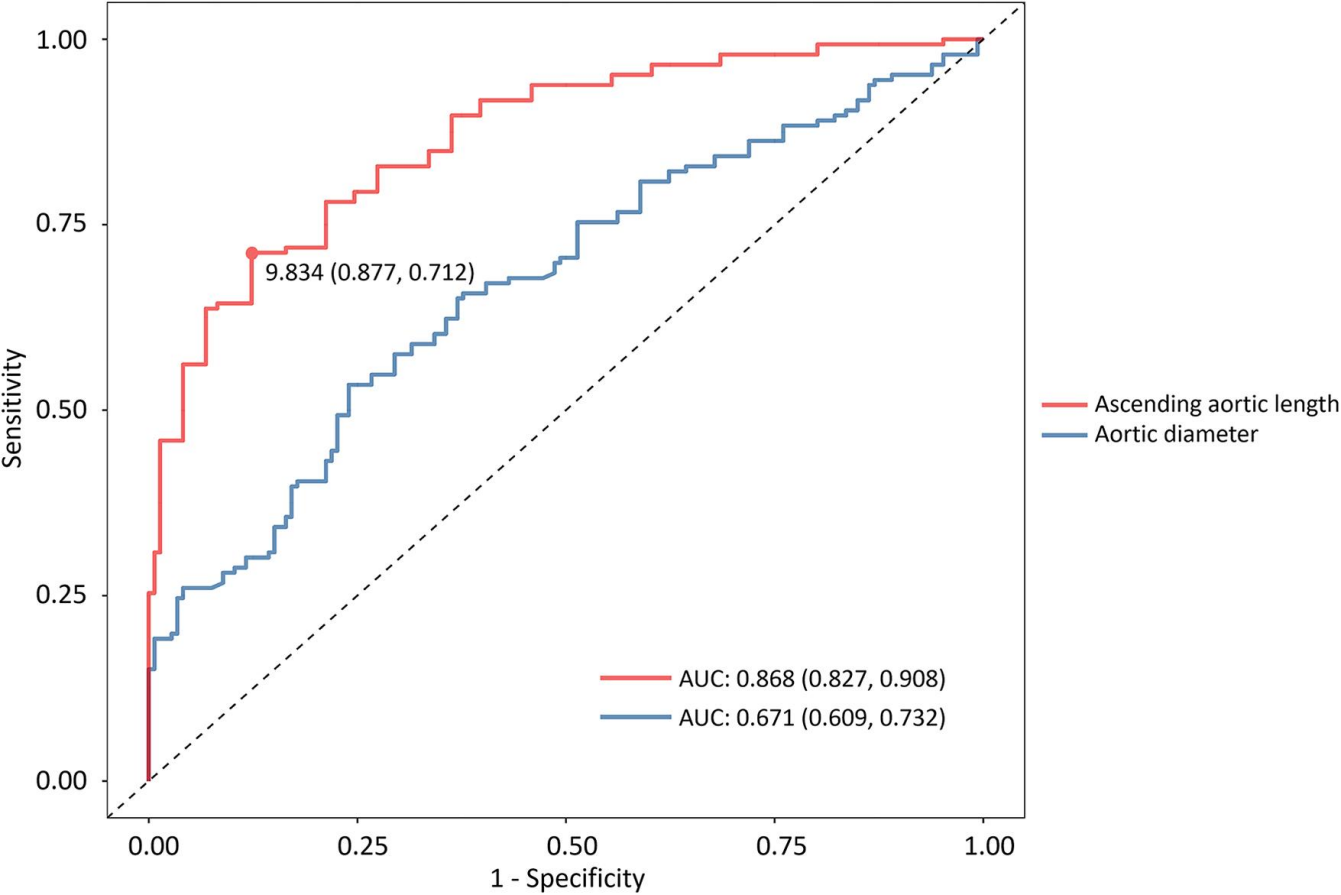
Receiver operating characteristic curves of two multivariate logistic regression models. The area under the curve demonstrates the predictive performance of the aortic diameter and AAL. AAL, ascending aortic length.

An analysis of the ROC curve of the AAL demonstrated an AUC of 0.868 (95% CI, 0.827–0.908) with a cutoff value of 9.8 cm to be associated with TAAD (sensitivity, 71.2%; specificity, 87.7%). In contrast, the ascending aortic diameter demonstrated an AUC of 0.671 (95% CI, 0.609–0.732) with a sensitivity of 53.4% and a specificity of 76% (Figure 4). Statistical comparison using the Delong test indicated a significant difference between the two AUCs (*P* < 0.001). In addition, internal bootstrap resampling (1,000 iterations) yielded consistent results, reinforcing the superior discriminative ability of the AAL (*P* < 0.001).

**Central Illustration:**
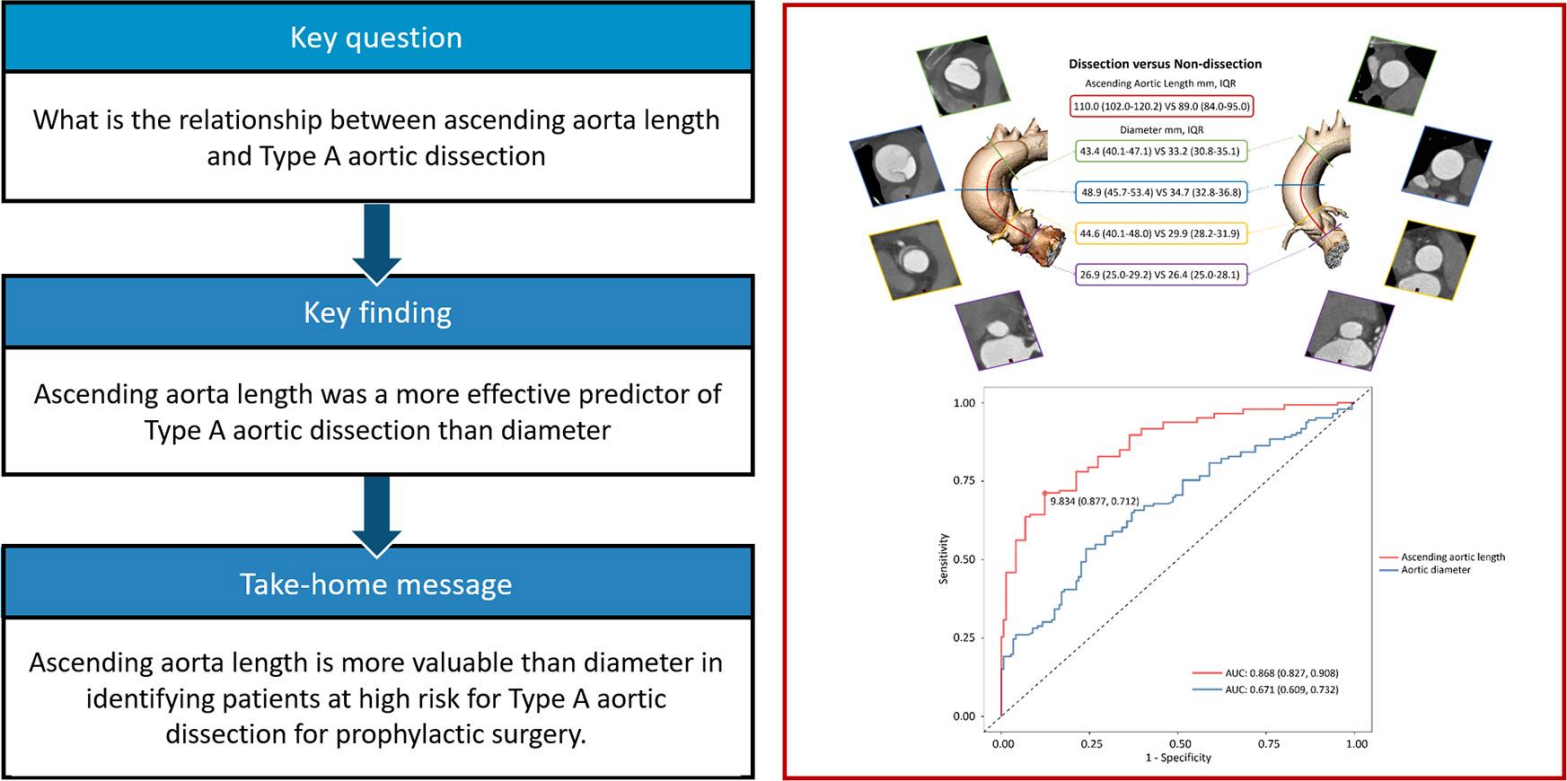
Aortic length performed better to predict type A aortic dissection compared with diameter.

## Discussion

In this study, we demonstrated an association between the ascending aortic elongation and TAAD independent of the aortic diameter in a Chinese population, among whom the aortic dimensions differ from those of the Western population. The predissection ascending aorta in patients with TAAD was 2.3 mm larger in diameter than that in the control participants. Although the predissection aortic diameter was significantly different between the two groups, the median aortic diameter of 37 mm indicated that most patients failed to meet the prophylactic operation criterion of 55 mm, which is in accordance with previous studies (3, 4, 12). In fact, only 22.6% of patients with TAAD met the indication criterion of diameter >55 mm in the present study. When adjusted for the predissection diameter, only one patient exhibited a diameter of >55 mm. Similar to previous studies, these results demonstrate that the aortic diameter alone is insufficient to act as the criterion for prophylactic replacement of the ascending aorta.

Notably, the predissection AAL was 15.4 mm longer in the patient group, indicating its potential to be applied as a morphometric risk factor for TAAD. When included in the multivariate logistic regression analysis, a 7.93-fold higher risk of TAAD was revealed with a 1-cm increase in the AAL. The results of the interaction and stratified analyses suggested that the AAL was associated with TAAD independent of age, sex, height, arch type, aortic diameter, hypertension, smoking history, dyslipidemia, and diabetes mellitus. The ROC curve showed a satisfactory AUC of 0.868 and presented an optimal cutoff value of 9.8 cm, which differs from the potential recommended criterion of 11 cm for a prophylactic operation (8). This difference may have arisen for two reasons. First, because of the rarity of acquiring both pre- and postdissection CTA images of the same patient, we used a cross-sectional observational study to investigate the association of the AAL and TAAD. However, the criterion of 11 cm was used in reference to a longitudinal cohort study. Second, the AAL in this study was shorter than that in previous studies, probably because of the different races of the study population. Furthermore, the model for estimating predissection aortic dimensions, derived from the Western population, may not be directly applicable to our Chinese cohort, highlighting the need for future studies to establish population-specific correction factors. Although the optimal cutoff value of 9.8 cm in this study may not be robust enough for direct recommendation in the clinical setting, the difference in the threshold values reminds us that the criterion of 11 cm generated from Western population data should not be indiscriminately used in the Chinese population.

In the ROC analysis, the AAL revealed better sensitivity and specificity in predicting the risk of TAAD than did the ascending aortic diameter when both were solely used, suggesting that the AAL may have better performance as a predictive factor of TAAD. This phenomenon can be explained from the perspectives of biomechanics and histology. In most cases, the aorta gradually expands both circumferentially and longitudinally before the onset of TAAD. A recent *in vitro* study that evaluated the aortic wall strength using normal human aortic tissue revealed a different degree of failure stress along the circumferential and longitudinal directions (13). Failure stress, which is the maximum value of stress the aortic tissue can resist, was twice as large in the circumferential direction than in the longitudinal direction. At the histological level, the media is the largest layer of the aortic wall that provides the main biomechanical strength. In the media, elastic fibers and collagen interconnect the elastic laminae, forming a continuous network with a three-dimensional helical structure in the circumferential direction (14). The orientation of these mural constituents theoretically provides a greater structural strength in the circumferential direction than in the longitudinal direction. In addition, horizontal intimal tears are commonly seen during operation in patients with TAAD (15, 16). With this understanding of different aortic wall strengths in the circumferential and longitudinal directions, we may assume that aortic elongation represents a chronic adaptive process that precedes and potentially facilitates subsequent circumferential expansion in the development of TAAD.

Other mechanisms related to aortic elongation also accelerate the development of TAAD. Aortic elongation is accompanied by extracellular matrix degradation with age, which compromises aortic integrity (17). Aortic elongation also leads to increased tortuosity of the ascending aorta, and the altered ascending aortic geometry will generate increased wall stress. The mechanotransduction pathways may be subsequently activated by the increased wall stress, which could lead to vascular smooth muscle cell apoptosis (18). While these pathophysiological insights are compelling, the precise temporal and causal relationship between aortic elongation and TAAD onset remains to be fully established. Future prospective studies are therefore warranted to longitudinally track aortic dimensions and definitively elucidate the role of ascending aortic elongation in the pathogenesis of TAAD.

A main advantage of the AAL as a predictive factor is its smaller variation after dissection onset. The recommended threshold of 5.5 cm was based on the dissected aortic diameter from an early study (2), in which the immediate variation of the diameter after dissection was not considered. Recent studies (5, 10, 12) have shown that the aortic diameter increases by 16.9%–31.9% when dissection occurs. However, these studies were based on CTA images within a certain period (such as 2 years) prior to dissection onset and immediately after aortic dissection. The predissection aortic diameter may be overestimated because of the difficulty of acquiring CTA images immediately before dissection onset. Even if the predissection aortic diameter is used to calculate a new cutoff value, a left-shift bias is inevitable. In contrast, in this study, the AAL increased by 2.7%–5.4% after dissection onset, which was much more stable. This relative stability of the AAL in aortic dissection could minimize the bias involved in calculating the intervention threshold.

Apart from these anatomical features, some other non-anatomical parameters are associated with aortic disease, including increased vessel wall stress, blood flow velocity, and inflammatory conditions. Looking ahead to the future clinical use of the AAL as a predictive factor of TAAD, a predictive model that combines other anatomical features and non-anatomical parameters is more likely to generate a better outcome. A specific and promising approach would be to develop a composite indicator that incorporates the AAL, diameter, and BSA, thereby capturing the complementary aspects of aortic geometry to achieve superior predictive performance. However, the complexity of such a predictive model would restrict its clinical application. Therefore, a CTA-based radiomics model may be a promising solution to enhance the applicability of a comprehensive predictive tool.

## Limitations

This study had several limitations. The first is its retrospective and cross-sectional observational nature. With this design, we were only able to demonstrate the association of the AAL and TAAD. To further determine the causal relationship between the AAL and TAAD, prospective longitudinal cohort studies are needed. Second, selection bias could not be prevented because we were not able to include patients who died before admission to the hospital. We also excluded patients with connective tissue disease or a bicuspid aortic valve. Third, we used estimated predissection aortic dimensions in the analysis, although the use of predissection aortic dimensions has been recommended in studies involving anatomical features of TAAD ever since this concept was proposed by Rylski et al. (5). Our application of their model—developed in a Western population—may introduce ethnic bias, as its accuracy in a Chinese population requires further validation. Fourth, our analysis did not incorporate the factor of normalization of aortic dimensions to the body surface area (BSA) or body mass index (BMI). It is well-established that aortic size scales with body habitus, and the use of size-indexed parameters (e.g., aortic size index) can improve risk stratification, as demonstrated in prior studies (8, 9). The decision to use absolute diameters in our primary analysis was made to facilitate direct clinical application and comparison with current guideline thresholds, which are predominantly based on absolute size. Nevertheless, we acknowledge that the absence of size-indexed metrics is a limitation, and future studies should evaluate the potential incremental value of such adjustments in this specific patient population with sporadic non-syndromic TAAD. Fourth, the relatively small sample size, particularly in subgroup analyses, resulted in wide confidence intervals, limiting the precision of our estimates. Finally, the predissection modeling itself entails uncertainty: the model relies on fixed coefficients from external data and does not account for variability in the timing between the last available imaging and the dissection event, which may affect accuracy.

## Conclusion

We demonstrated that the AAL is independently associated with TAAD. When both were solely used, the AAL was more promising as a predictive factor of sporadic, non-syndromic TAAD than was the aortic diameter. Observed AAL values associated with TAAD were lower in the Chinese cohort than in the Western population. To further determine a convincing intervention threshold of the AAL, a prospective longitudinal study with a large sample size is required to be conducted.

## Data Availability

The original contributions presented in the study are included in the article/Supplementary Material, further inquiries can be directed to the corresponding author.
